# Exploring the pathogenic mechanisms of dementia risk factors: a task-fMRI study of mild cognitive impairment

**DOI:** 10.3389/fnins.2026.1751092

**Published:** 2026-02-24

**Authors:** Yuling Shen, Min Shi, Shanyu Liu, Yan Liu, Lijun Wang, Dongdong Yang

**Affiliations:** 1 Department of Neurology, Shenzhen Hospital (Futian) of Guangzhou University of Chinese Medicine, Shenzhen, China; 2 Department of Neurology, Hospital of Chengdu University of Traditional Chinese Medicine, Chengdu, China; 3 Department of Integrated Traditional Chinese and Western Medicine, West China Hospital, Sichuan University, Chengdu, China

**Keywords:** mild cognitive impairment, task based functional magnetic resonance imaging, risk factors, brain activity, default mode network

## Abstract

**Objective:**

To investigate the mechanism by which risk factors influence brain functioning using task-based functional magnetic resonance imaging (fMRI), providing a theoretical basis for controlling these risk factors.

**Methods:**

Risk factors associated with MCI-to-AD conversion were collected from 31 amnestic mild cognitive impairment (aMCI) patients and scored according to the Cardiovascular Risk Factors, Aging, and Dementia (CAIDE) dementia risk scale. The relationships between risk scores, cognitive function, and task-based fMRI brain activity were analyzed.

**Results:**

Risk factor score was negatively correlated with multiple cognitive performances, including the Mini-Mental State Examination (MMSE), Montreal Cognitive Assessment (MoCA), Auditory Verbal Learning Test (AVLT) immediate recall and delayed recall, digit span forward and backward, and Boston Naming Test (BNT). Task-based fMRI whole-brain and region-of-interest (ROI) analyses revealed a positive correlation between risk factor score and brain activity in default mode network (DMN) during the retrieval phase.

**Conclusion:**

Risk factors can abnormally increase brain activity in DMN. Given the close association between amyloid-β (Aβ) deposition and reduced DMN deactivation, these risk factors may elevate DMN activity, thereby facilitate Aβ accumulation in these regions.

## 1 Introduction

MCI is an intermediate state between normal cognition and dementia, characterized by impairment in one or more cognitive domains without affecting daily living abilities, and not meeting the diagnostic criteria for dementia (Chukwujama and Gormley, 2011). Study has found that conversion risk of MCI is 41.5% in clinical and 27.0% in population-based studies (Salemme et al., 2025). Multiple factors, such as advanced age (Chen et al., 2018), diabetes (Cooper et al., 2015), hypertension, and hyperlipidemia (Solfrizzi et al., 2011; Li et al., 2011), can accelerate the progression from MCI to AD. Several studies have developed dementia risk prediction scores based on these risk factors (Kivipelto et al., 2006; Barnes et al., 2009; Reitz et al., 2010), including the CAIDE dementia risk score from Finland. Since the risk factors it incorporates coincide with those influencing the progression of MCI and it demonstrates good predictive performance, we adopted the CAIDE dementia risk score to quantify these risk factors and investigate the mechanisms by which they influence brain functioning.

Recent advances in neuroimaging have not only enabled scientists and clinicians to detect Alzheimer’s disease and related dementia pathology in the brains of living adults, but also serve as a prognostic tool to help predict cognitive decline and identify individuals at increased risk of progression from MCI to Alzheimer’s disease AD (Iaccarino et al., 2017; O'Brien et al., 2010; Smailagic et al., 2018; Vecchio et al., 2025). In recent years, task-based fMRI, which reflects changes in brain function, has gained increasing attention. fMRI studies the neural basis of higher cognitive functions by measuring regional hemodynamic changes related to underlying cellular activity, known as blood oxygen level dependent (BOLD) imaging. Task-based fMRI refers to the acquisition of brain images while participants perform specific tasks during scanning. It typically involves comparing BOLD signals under a task condition (e.g., memory encoding of novel stimuli) with those under a control condition (e.g., viewing familiar stimuli) or a passive baseline condition (e.g., visual fixation). An increase in the BOLD signal indicates activation of the corresponding brain region, whereas a decrease indicates deactivation. Task-based fMRI can identify changes in the activity of memory-related brain regions in AD, AD-related MCI, and even preclinical AD, and has been extensively applied in studies of AD and MCI.

The memory process is supported by a complex network of distributed, large-scale brain regions, including the hippocampus and a set of cortical regions collectively referred to as the DMN (Raichle et al., 2001; Buckner et al., 2008). The hippocampus plays a critical role in memory by encoding the places and events that constitute episodic memory and linking them together through their shared elements (Eichenbaum, 2000). fMRI studies using memory tasks in both young and older participants (Sperling et al., 2001, 2003) have demonstrated hippocampal hyperactivation during memory process. Successful memory formation depends not only on the activation of memory-related brain regions but also on the selective deactivation or inhibition of irrelevant regions. The fundamental mechanism of this inhibition may involve suppressing cortical regions unrelated to memory in order to facilitate the activation of memory-related regions. In other words, unnecessary sensory inputs are suppressed to improve efficiency, allowing relevant information to be processed effectively. Task-based fMRI studies have revealed that the DMN is active during rest but deactivates during memory process, and such deactivation is associated with successful memory encoding (Buckner et al., 2008). Task-based fMRI further indicates that deactivation of key DMN nodes coordinated with hippocampal activation is a prerequisite for successful memory encoding (Miller et al., 2008). Current task-based fMRI research on the AD continuum primarily focuses on brain activity within the hippocampus and the DMN.

In this study, dementia risk factors were quantified using the Finnish CAIDE dementia risk score to investigate their associations with brain activity in hippocampus and the DMN during the memory encoding and retrieval stages. By elucidating the mechanisms through which these risk factors influence brain functioning, the study aims to provide a theoretical basis for early intervention targeting modifiable risk factors, which holds important clinical significance.

## 2 Materials and methods

### 2.1 Participants

This study recruited individuals with aMCI who were admitted to the Hospital of Chengdu University of TCM (Chengdu, China) from November 2022 to April 2024. Written informed consent was obtained from all participants prior to their participation. Ethical approval was granted by the Medical Ethics Committee of the Hospital of Chengdu University of TCM (Approval No. 2022KL-042-02).

Inclusion criteria were as follows: (1) Participants were required to be 50–80 years old. (2) Right-handed. (3) Sufficient audiovisual and comprehension abilities to complete neuropsychological assessments and task-based functional MRI. (4) Diagnosed with aMCI, characterized by memory decline persisting for over 6 months, a global Clinical Dementia Rating score of 0.5, and long-term memory impairment, defined as scoring at least one standard deviation below the age-adjusted normative mean on the Auditory Verbal Learning Test–HuaShan version (AVLT) (Guo et al., 2009; Zhang et al., 2014). Specifically, the delayed recall score thresholds on the AVLT were ≤5 for participants aged 50–59, ≤4 for those aged 60–69, and ≤3 for those aged 70–79 (Albert et al., 2011; Jack et al., 2018). The aMCI diagnosis was ascertained by researchers trained in standardized assessment protocols.

Exclusion criteria: (1) comorbid conditions such as tumors, severe cardiac, liver, kidney, or hematological disorders, psychiatric illnesses, and medical conditions associated with cognitive impairment (e.g., cerebrovascular disease, Parkinson’s syndrome, normal pressure hydrocephalus, vitamin B1 or B12 deficiency, thyroid dysfunction, alcoholism, or carbon monoxide poisoning). (2) Severe visual or hearing impairments. (3) A history of drug or alcohol abuse. (4) MRI contraindications, including pacemakers, metal implants, or claustrophobia. (5) A Hamilton Depression Scale score above 17, and (6) the participant’s functional MRI images are of poor quality and cannot be used for analysis.

### 2.2 Neuropsychological assessment

The MMSE and MoCA were used to assess global cognitive function. The AVLT assessed episodic memory. The Digit Span Test assessed working memory. The Boston Naming Test and Verbal Fluency Test assessed language function. The Clock Drawing Test assessed visuospatial function. The Clinical Dementia Rating assessed the severity of cognitive impairment. The Activities of Daily Living scale assessed daily living activities. The Hamilton Depression Rating Scale assessed depressive status to exclude depressed patients.

### 2.3 Risk factors collection

Participants underwent collection of general information and vascular risk factors. General information included sex, age, height, weight, and years of education, while vascular risk factors included hypertension, dyslipidemia, and physical activity. Hypertension and dyslipidemia were defined based on participants’ self-reported medical history or use of relevant medications, and verified through their medical records. Participants reporting a history of hypertension and having documented hypertension in their medical records or currently taking antihypertensive medications were classified as having hypertension. Similarly, participants reporting dyslipidemia and with documented dyslipidemia in their medical records or currently taking lipid-lowering medications were classified as having dyslipidemia. Body mass index (BMI) was calculated from height and weight, with BMI exceeding 30 kg/m^2^ considered overweight. Physical activity was defined based on the frequency of leisure-time exercise. Participants engaging in physical activity at least twice per week, for 20–30 min per session, and inducing sweating were classified as active; all others were classified as inactive.

### 2.4 Risk factor scoring

Risk factors were scored according to the Finnish CAIDE dementia risk score (Barnes et al., 2009). Age was categorized as 50–53 years (3 points), and older than 53 years (4 points). Education was scored as more than 10 years (0 points), 7–9 years (2 points), and 0–6 years (3 points). Sex was assigned 0 points for females and 1 point for males. Hypertension was scored 2 points if present and 0 points if absent. BMI was assigned 2 points for values exceeding 30 kg/m^2^ and 0 points for values of 30 kg/m^2^ or less. Dyslipidemia was scored 2 points if present and 0 points if absent. Physical activity was scored 0 points for participants who were physically active and 1 point for those who were inactive. The scores assigned to each individual risk factor are ultimately summed to generate an overall composite risk score.

### 2.5 Episodic memory task

Task-based fMRI assesses changes in brain activity while subjects perform tasks. As aMCI patients primarily exhibit memory impairment, an event-related vocabulary memory task was developed and programmed using E-Prime 3.0 (Psychology Software Tools, Inc., Pittsburgh, USA). Stimuli were presented to subjects via an magnet-compatible brain function audiovisual stimulation system (SA-9939; Sinorad Medical Electronics Co., Ltd., Shenzhen, China), and behavioral data were acquired via a subject response feedback and synchronization system.

The vocabulary memory task consisted of 3 runs. Each run contained two vocabulary encoding blocks alternating with two vocabulary retrieval blocks. During the encoding phase, 15 words were presented on the screen and participants were instructed to memorize the words. During the retrieval phase, 30 words were presented, consisting of 15 previously encoded words and 15 novel ones, and participants were instructed to determine whether the words were old or new. Words were presented for 1 s each, with inter-stimulus intervals ranging from 2 to 8 s. Participants were given a detailed briefing on the examination procedure 30 min prior to the scan and completed practice tasks on a laptop that differed from the formal trial.

### 2.6 Task-based fMRI acquisition

Task-based fMRI scanning was performed using Echo-Planar Imaging with the following parameters: Repetition Time = 2000 ms, Echo Time = 30 ms, flip angle = 90°, voxel size = 3.75 mm × 3.75 mm × 3.5 mm, field of view = 240 mm × 240 mm, matrix = 64 × 64, slice thickness = 3.5 mm, no gap, number of slices = 39.

### 2.7 Preprocessing

Task-based fMRI data were preprocessed and analyzed using the Statistical Parametric Mapping 12 (SPM12) software package. To eliminate the effects of magnetic field instability at the beginning of scanning, the first 10 volumes of each task-based fMRI dataset were discarded. Preprocessing in SPM12 included the following steps: (1) slice timing correction, which is applied to correct temporal differences between sequentially acquired slices. (2) Realignment: all images were aligned to the first image to correct for head motion during scanning. (3) Spatial normalization: Images were normalized to the Montreal Neurological Institute template for group-level analysis, followed by reslicing the normalized images to a voxel size of 3 × 3 × 3 mm^3^; and (4) smoothing: Spatial smoothing with a 6 mm FWHM Gaussian kernel was applied to improve signal-to-noise ratio and account for anatomical variability.

### 2.8 Data analysis

Based on whether participants responded correctly during the retrieval phase, memory stimuli were categorized into four types: correct encoding, incorrect encoding, correct retrieval, and incorrect retrieval. A general linear model of the hemodynamic responses for the aforementioned four types is constructed using the HRF. Temporal derivatives were included in the model, and the six motion parameters (three translations, three rotations) were added as covariates. Statistical parameter estimates for the different types were then derived based on the model. To investigate brain activity during the encoding and recognition phases, contrasts were defined as follows: (1) encoding phase: correct encoding vs. baseline; (2) retrieval phase: correct recognition vs. baseline. Contrast maps were created for each subject.

To investigate the relationship between brain activity and risk factors in patients with MCI during the encoding and recognition phases, whole-brain multiple regression analyses were conducted using SPM12. Brain activity during the encoding or recognition phase in MCI patients was treated as the dependent variable, and risk factor scores were included as the independent variable, with sex, age, and education included as covariates. Whole-brain analyses were performed with a statistical threshold of *p* < 0.005 and an extent threshold of 10 contiguous voxels, meaning that only clusters with at least 10 contiguous voxels were considered significant.

ROI analyses were conducted using hippocampus and DMN masks constructed with MarsBaR. The DMN mask encompasses brain regions including the posterior cingulate cortex, precuneus, and medial prefrontal cortex. Multiple regression analyses were performed in SPM12 to examine the relationship between brain activity in hippocampus and DMN and risk factors. Brain activity within the ROI during the encoding or recognition phase in MCI patients was treated as the dependent variable, with risk factor scores as the independent variable, controlling for sex, age, and education. Statistical significance was defined at *p* < 0.01 with an extent threshold of 10 contiguous voxels, meaning that only clusters with at least 10 contiguous voxels were considered significant.

### 2.9 Statistical analysis

Statistical analyses were performed using SPSS 26.0 software. Continuous variables with a normal distribution were presented as mean ± standard deviation, whereas non-normally distributed continuous variables were reported as median (interquartile range, IQR). Categorical variables were expressed as counts and percentages. As the risk factor scores were not normally distributed, Spearman correlation analyses were conducted to examine the associations between risk factor scores and cognitive performance.

## 3 Results

### 3.1 General characteristics

A total of 40 individuals with aMCI participated in the study. Of these, 2 participants withdrew from the study and declined to complete the questionnaires and functional MRI assessments; 3 participants could not tolerate the relatively long duration of the functional MRI scan; 2 participants were excluded due to excessive head motion that compromised image quality; and 2 participants were excluded because they failed to complete the memory task during the functional MRI procedure. Consequently, a total of 31 participants were included in the final analysis. The general characteristics of the aMCI group are summarized in Table 1.

**Table 1:** Participants’ general characteristics.

Items	aMCI group (*n* = 31)
Age (years), mean (SD)	58.55 (6.31)
Gender, female, *n* (%)	46 (76.7%)
Education (years)	8 (6,11.5)
Physical activity, *n* (%)	13 (21.7%)
BMI (Kg/m^2^), mean (SD)	23.57 (0.37)
Hypertension, *n* (%)	6 (10%)
Dyslipidemia, *n* (%)	6 (10%)
MMSE	26 (25, 27)
MoCA, mean (SD)	21.11 (0.57)
AVLT (immediate recall), mean (SD)	12.35 (0.54)
AVLT (delayed recall)	3 (2.75, 4)
AVLT (recognition)	19 (17, 20.25)
Digit span forward	7 (6, 8)
Digit span backward	4 (3, 4)
Boston Naming Test, mean (SD)	20.35 (0.56)
Animal Fluency Test, mean (SD)	12.30 (0.36)
Clock Drawing Test	3 (2, 4)

aMCI, amnestic mild cognitive impairment; SD, standard deviation; BMI, body mass index; MMSE, Mini-Mental State Examination; MoCA, Montreal Cognitive Assessment; AVLT, Auditory Verbal Learning Test.

### 3.2 Correlation between risk factor score and cognitive performance

**Figure 1:**
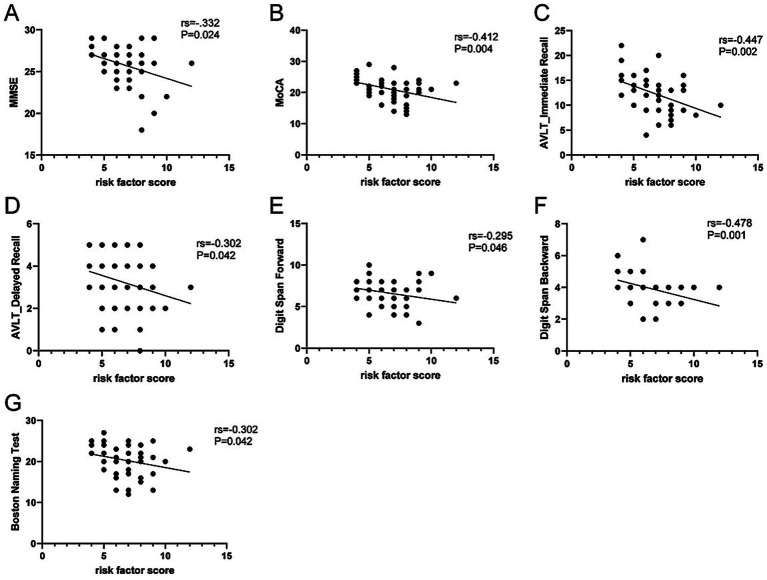
Scatter plot illustrating the association between risk factor score and neuropsychological test scores. **(A)** Correlation between MMSE score and risk factor score, *rs* = −0.332; **(B)** Correlation between MoCA score and risk factor score, *rs* = −0.412; **(C)** Correlation between AVLT immediate recall score and risk factor score, *rs* = −0.447; **(D)** Correlation between AVLT delayed recall score and risk factor score, *rs* = −0.302; **(E)** Correlation between digit span forward score and risk factor score, *rs* = −0.295; **(F)** Correlation between digit span backward score and risk factor score, *rs* = −0.478; **(G)** Correlation between Boston Naming Test score and risk factor score, *rs* = −0.302. *p* < 0.05.

As the risk factor scores were not normally distributed, spearman correlation analysis was conducted to examine the association between risk factor scores and cognitive performance. Risk factor score was negatively correlated with MMSE score, MoCA score, AVLT immediate recall score, AVLT delayed recall scores, digit span forward scores, digit span backward scores, and BNT scores. Detailed results are presented in Table 2, Figures 1A–G.

**Table 2:** Correlations between risk factor score and cognitive performance.

Neuropsychological test scores	Risk factor score	
	*p*	*rs*
MMSE	0.0241	−0.332^*^
MOCA	0.004	−0.412^*^
AVLT (immediate recall)	0.002	−0.447^*^
AVLT (delayed recall)	0.042	−0.302^*^
AVLT (recognition)	0.293	−0.159
Digit span forward	0.046	−0.295^*^
Digit span backward	0.001	−0.478^*^
Boston Naming Test	0.042	−0.302^*^
Animal Fluency Test	0.129	−0.227
Clock Drawing Test	0.07	−0.269

MMSE, Mini-Mental State Examination; MoCA, Montreal Cognitive Assessment; AVLT, Auditory Verbal Learning Test. ^*^p < 0.05 indicates statistical significance.

### 3.3 Relationship between risk factor score and task-based fMRI brain activity in whole-brain analysis

**Figure 2:**
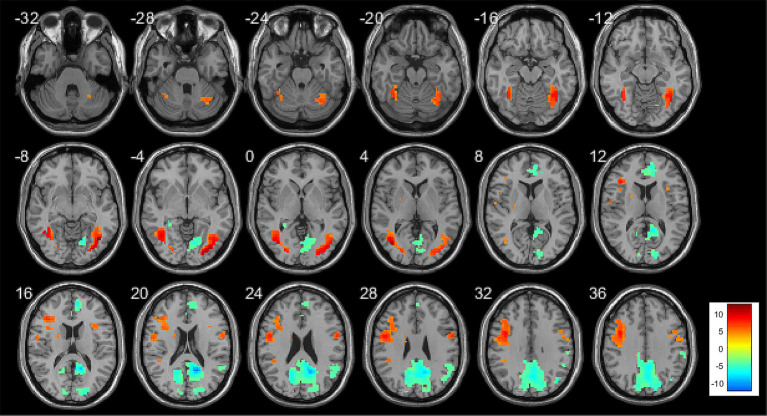
Brain activation map of aMCI patients during the encoding phase, where yellow indicates activation (correct encoding minus baseline) and green indicates deactivation (baseline minus correct encoding). Threshold: *p* < 0.001, cluster size >10 voxels.

**Figure 3:**
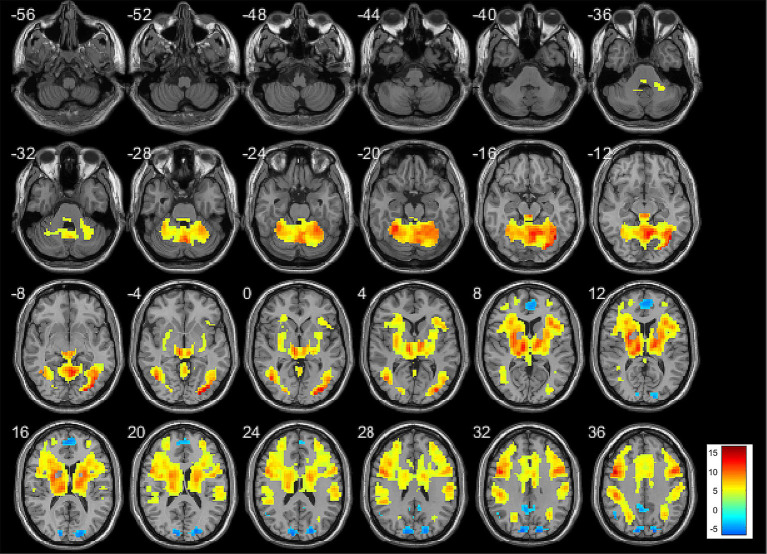
Brain activation map of aMCI patients during the retrieval phase, where yellow–red indicates activation (correct recognition minus baseline) and blue indicates deactivation (baseline minus correct recognition). Threshold: *p* < 0.001, cluster size >10 voxels.

**Figure 4:**
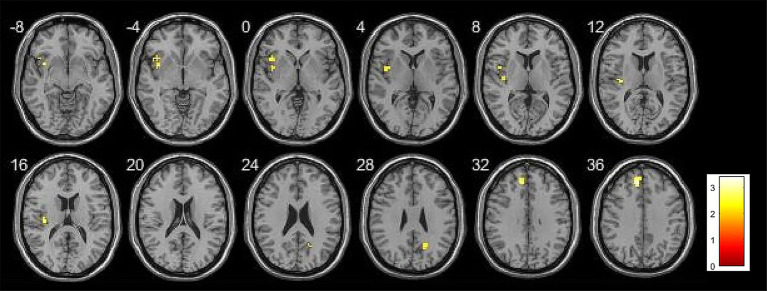
Brain activation map showing the association between risk factor score and brain activity during retrieval phase in whole brain analysis. Threshold: *p* < 0.005, cluster size >10 voxels.

The regions of activation and deactivation in aMCI patients during memory task performance are shown in Figures 2, 3. Whole-brain analysis was conducted to examine the relationship between risk factor score and memory task-related brain activity. During the encoding phase, no significant correlations were observed between risk factor score and brain activity. During the retrieval phase, risk factor score was positively correlated with activity in the left dorsolateral superior frontal gyrus, left insula, left precentral gyrus, left triangular part of the inferior frontal gyrus, and right precuneus. Detailed results are presented in Table 3, Figure 4.

**Table 3:** Brain regions showing memory-induced activity correlated with risk factor score in whole brain analysis (threshold *p* < 0.005, cluster size >10 voxels).

Anatomical region	BA	Peak coordinates (MNI)			*T* value	Cluster size
		*X*	*Y*	*Z*		
Correct retrieval-baseline						
Positive correlation						
Left dorsolateral superior frontal gyrus		−12	39	36	4.58	37
Left insula	13	−39	9	−6	3.65	29
Left parietal operculum	13	−39	−18	12	3.43	13
Left inferior frontal gyrus, triangular part		−45	18	0	3.38	18
Right precuneus		18	−60	27	3.26	10

BA, Brodmann area.

### 3.4 Relationship between risk factor score and task-based fMRI brain activity in roi analysis

**Figure 5:**
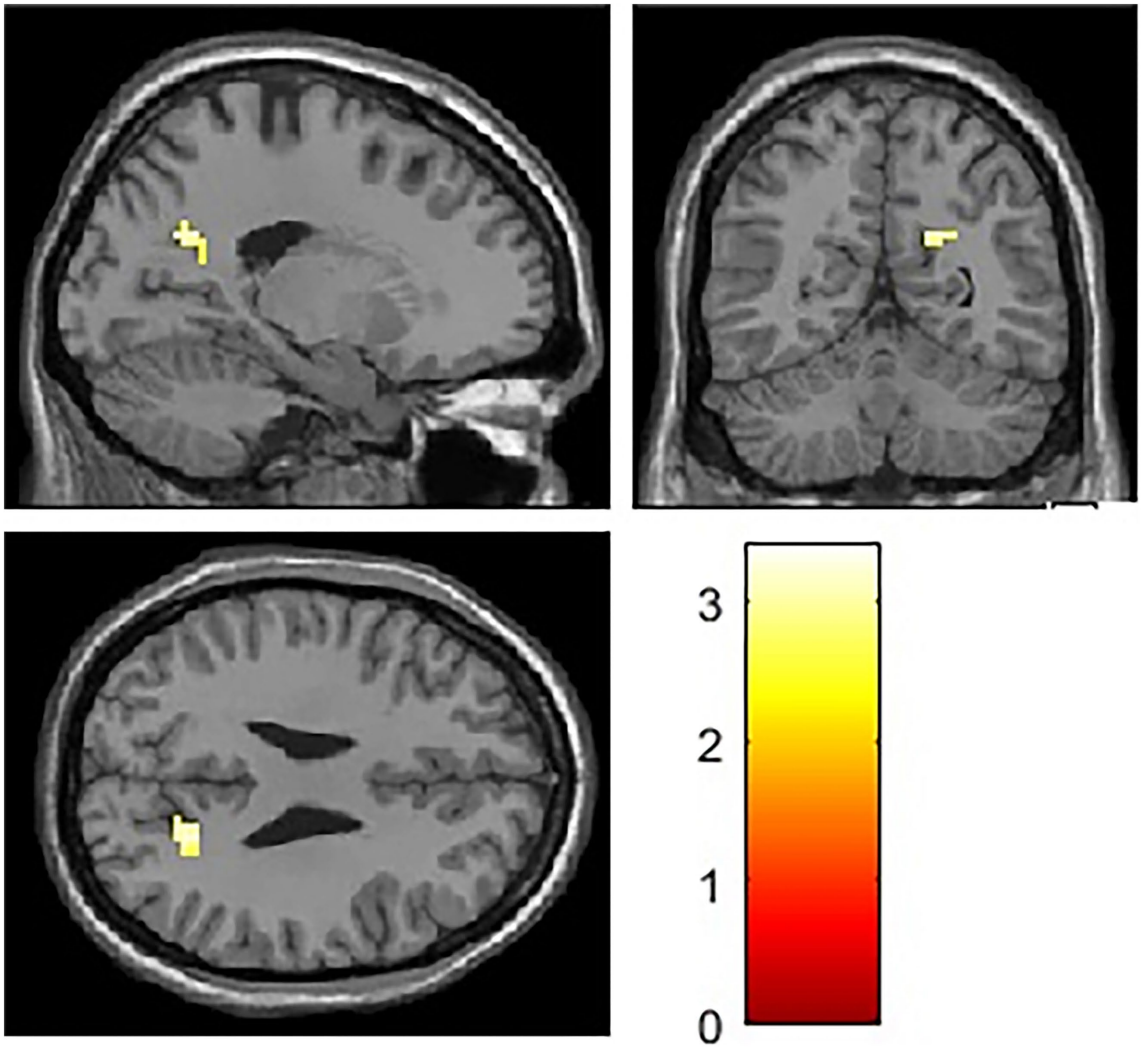
Brain activation map showing the association between risk factor score and brain activity during retrieval phase in ROI analysis. Threshold: *p* < 0.01, cluster size >10 voxels.

**Figure 6:**
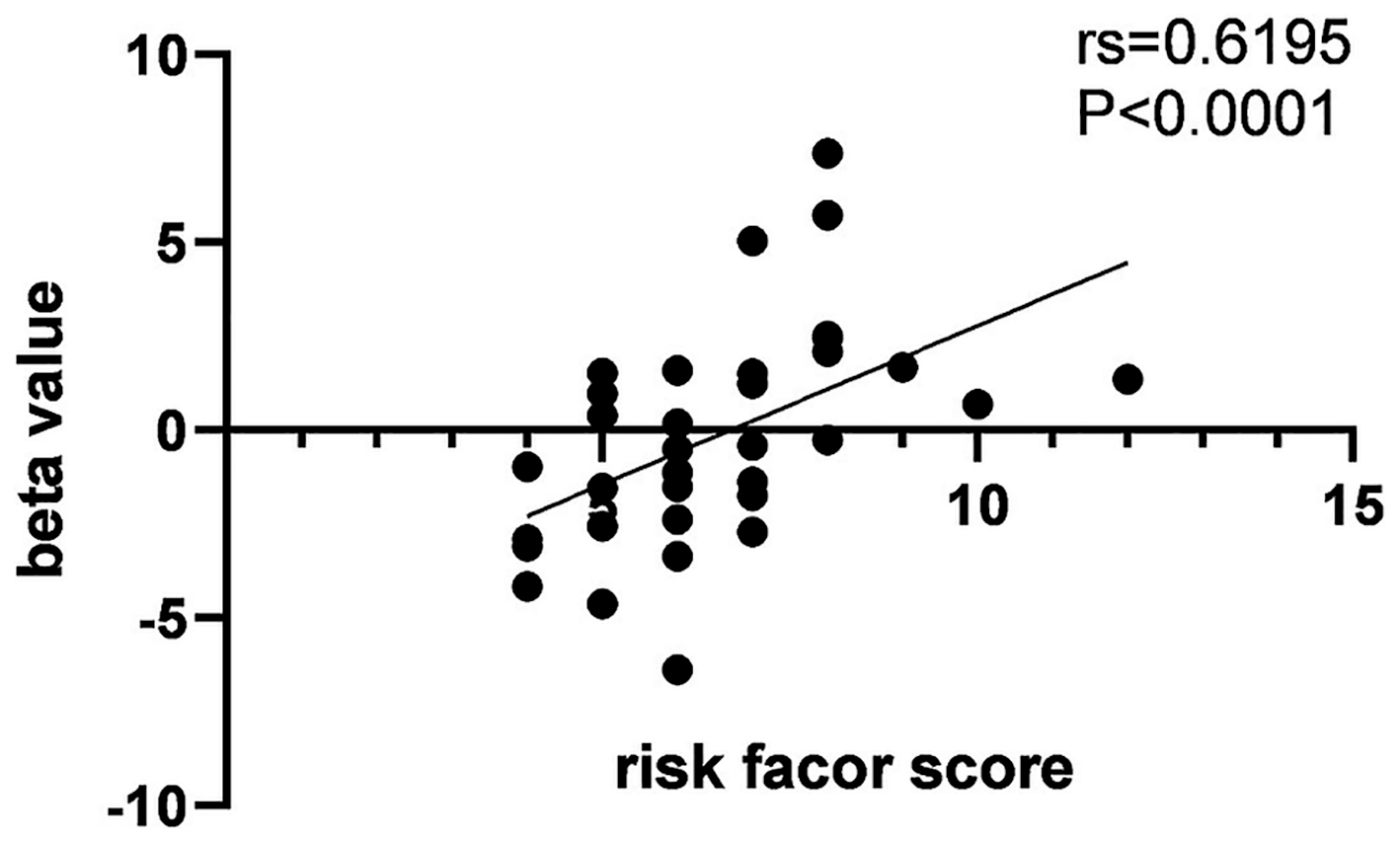
Scatter plot illustrating the association between risk factor score and brain activity in the ROI analysis. *rs* = 0.6195, *p* < 0.0001.

ROI analysis was conducted to investigate the association between risk factor score and memory task-induced brain activity within the hippocampus and DMN. During the encoding phase, no significant correlations were observed between risk factor score and brain activity in DMN. During the retrieval phase, risk factor score was positively correlated with activity in the right precuneus. Detailed results are presented in Table 4, Figures 5, 6.

**Table 4:** Brain regions showing memory-induced activity correlated with risk factor score in ROI analysis (threshold *p* < 0.01, cluster size >10 voxels).

Anatomical region	BA	Peak coordinates (MNI)			*T* value	Cluster size
		*X*	*Y*	*Z*		
Correct retrieval-baseline						
Positive correlation						
Right precuneus		12	−66	36	3.39	34

BA, Brodmann area.

## 4 Discussion

This study initially explored the relationship between risk factor score and cognitive performance, finding that higher score was associated with poorer cognitive performance. Although no previous studies have directly examined the association between a composite risk factor score and cognitive performance, several studies have shown that controlling individual risk factors can delay cognitive deterioration (Baumgart et al., 2015; Ngandu et al., 2015), indirectly supporting the negative impact of these risk factors on cognitive performance.

In this study, multiple factors contributing to the progression of MCI were quantified to investigate the relationships between risk factor score and brain activity measured by fMRI, thereby elucidating the potential mechanisms underlying their effects. Both whole-brain and ROI analyses revealed that, during the retrieval phase, risk factor score was positively correlated with brain activity in DMN.

The results of this study are consistent with those of previous research; however, unlike earlier studies that included cognitively normal individuals, this study focused on participants with MCI. Chen et al. (2016) and Marder et al. (2014) examined differences in brain activity between healthy controls and individuals with type 2 diabetes, both reporting increased activation in DMN-related regions in the diabetic group. Similarly, McDonough et al. (2019) quantified risk factors in HC participants and examined the association between risk score and brain activity, showing that higher dementia risk score was positively correlated with brain activity in DMN.

Previous research in AD has mapped the early “topography” of pathological changes in the brain (Braak et al., 2011). Aβ plaques initially emerge in the neocortex, particularly in the medial prefrontal and midline parietal regions, which largely overlap with the DMN. Tau tangles originate in the medial temporal lobe, initially affecting the entorhinal cortex, these vulnerable regions also correspond to areas showing early hypometabolism (Klunk et al., 2004) and atrophy in AD (Buckner et al., 2005). Models of AD progression propose that an overburdened neural system may facilitate the accumulation of AD-related pathology, (Buckner et al., 2009; Jones et al., 2016) where prolonged high-load neural activity leads to “network exhaustion” and, in some individuals, triggers the AD cascade.

The DMN plays a critical role in memory, being active during rest and deactive during memory tasks to support successful encoding. DMN deactivation is closely associated with the success of memory encoding (Buckner et al., 2008). In the present study, higher risk factor score was associated with increased activity in DMN, suggesting that risk factors may induce hyperactivity or overloading of DMN networks.

Several studies have reported that Aβ initially accumulates in DMN regions, particularly in critical region like the precuneus (Braak et al., 2011; Aizenstein et al., 2008; Rodrigue et al., 2012). Buckner et al. (2009) and Sperling et al. (2009) found that elevated Aβ deposition was associated with increased activity in DMN among cognitively normal individuals and participants with MCI. We hypothesize that risk factors may impose additional load on DMN, disrupt the balance between neuroprotective and neurotoxic microenvironments within the brain, ultimately promote Aβ deposition in these regions and facilitate its subsequent spread to other regions of the brain.

Several studies have explored the relationship between blood lipids and Aβ deposition. Reed et al. (2014) and Nägga et al. (2018) reported that higher blood lipid levels were associated with Aβ accumulation in both cognitively normal individuals and those with MCI, providing partial support for our hypothesis. In contrast, Cheng et al. (2020) and Vemuri et al. (2017) examined the relationship between multiple risk factors and Aβ aggregation, finding that only blood lipids level was related to Aβ deposition, while other factors such as hypertension, hyperglycemia, and obesity showed no significant associations. Therefore, in future studies, we plan to investigate the relationships between composite risk factor score and Aβ deposition to further validate the mechanisms proposed in the present study.

This study has several limitations. First, although we investigated the relationship between risk factor score associated with MCI progression and brain activity measured by fMRI, not all potential risk factors were included in the dementia risk score. We used the CAIDE dementia risk score developed in Finland, which incorporates age, sex, blood pressure, BMI, education, blood lipids, and physical activity, but does not account for genetic information, family history, smoking, or alcohol consumption. Second, increases in the BOLD signal are typically considered indicative of elevated neuronal activity in the corresponding brain regions. However, advanced age and cerebrovascular risk factors may disrupt the coupling between BOLD signals and neuronal activity (Mohtasib et al., 2012). We did not calibrate the BOLD signal to account for such confounding effects, which may have inflated the association between risk factors and brain activity. Moreover, in this study, the whole-brain analysis did not apply strict correction for multiple comparisons in order to explore potential patterns of brain activation and retain more possible significant signals. However, a stringent threshold was set. Additionally, ROI analysis was conducted. Both the whole-brain and ROI analyses revealed a positive correlation between risk factors and the right precuneus within the default mode network, supporting the relative reliability of this finding. However, other brain regions identified in the whole-brain analysis as related to risk factors may include false-positive results. Future studies with larger sample sizes and the use of multiple comparison correction methods (e.g., FWE, FDR) are needed to confirm the robustness of the other whole-brain findings. Finally, during episodic memory task, both correct encoding and correct retrieval are thought to engage memory-related brain regions and induce corresponding hemodynamic changes. Accordingly, this study used the contrasts of correct encoding vs. baseline and correct retrieval vs. baseline to examine alterations in memory-related brain activity and to further explore the relationship between risk factors and memory-related neural responses. However, the present study did not further examine brain activity changes or their associations with risk factors using additional contrasts such as incorrect encoding vs. baseline, incorrect retrieval vs. baseline, correct encoding vs. incorrect encoding, or correct retrieval vs. incorrect retrieval. Future studies will incorporate these contrasts to clarify whether the effects observed here are specific to successful memory performance, thereby strengthening the claims regarding the specificity of risk factor effects on memory-related brain activity and enhancing the overall rigor and interpretability of the findings.

## 5 Conclusion

Risk factors can negatively impact cognitive performance. Both whole-brain and ROI analyses of task-based fMRI revealed positive correlation between risk factor score and brain activity in DMN. Given the close relationship between elevated DMN activity and Aβ deposition, these findings indicate that risk factors may promote Aβ accumulation in DMN regions.

## Data Availability

The original contributions presented in the study are included in the article/supplementary material, further inquiries can be directed to the corresponding author.
